# Lateral and feedforward inhibition suppress asynchronous activity in a large, biophysically-detailed computational model of the striatal network

**DOI:** 10.3389/fncom.2014.00152

**Published:** 2014-11-25

**Authors:** Jason T. Moyer, Benjamin L. Halterman, Leif H. Finkel, John A. Wolf

**Affiliations:** ^1^Department of Bioengineering, University of PennsylvaniaPhiladelphia, PA, USA; ^2^Department of Neurosurgery, University of PennsylvaniaPhiladelphia, PA, USA

**Keywords:** striatum, computational model, basal ganglia, inhibition, signal-to-noise ratio, Tourette syndrome, Parkinson disease, obsessive-compulsive disorder

## Abstract

Striatal medium spiny neurons (MSNs) receive lateral inhibitory projections from other MSNs and feedforward inhibitory projections from fast-spiking, parvalbumin-containing striatal interneurons (FSIs). The functional roles of these connections are unknown, and difficult to study in an experimental preparation. We therefore investigated the functionality of both lateral (MSN-MSN) and feedforward (FSI-MSN) inhibition using a large-scale computational model of the striatal network. The model consists of 2744 MSNs comprised of 189 compartments each and 121 FSIs comprised of 148 compartments each, with dendrites explicitly represented and almost all known ionic currents included and strictly constrained by biological data as appropriate. Our analysis of the model indicates that both lateral inhibition and feedforward inhibition function at the population level to limit non-ensemble MSN spiking while preserving ensemble MSN spiking. Specifically, lateral inhibition enables large ensembles of MSNs firing synchronously to strongly suppress non-ensemble MSNs over a short time-scale (10–30 ms). Feedforward inhibition enables FSIs to strongly inhibit weakly activated, non-ensemble MSNs while moderately inhibiting activated ensemble MSNs. Importantly, FSIs appear to more effectively inhibit MSNs when FSIs fire asynchronously. Both types of inhibition would increase the signal-to-noise ratio of responding MSN ensembles and contribute to the formation and dissolution of MSN ensembles in the striatal network.

## Introduction

The basal ganglia, a set of interconnected neural structures positioned deep in the brain, are believed to be critically involved in learning (Graybiel, 1995; Houk et al., 1995; Pasupathy and Miller, 2005), motivation (Salamone and Correa, 2002; Yin and Knowlton, 2006), action selection (Gurney et al., 2001a,b; Humphries et al., 2006; Nicola, 2007), and motor control (Mink, 1996; Doya, 2000; Hikosaka et al., 2000; Turner and Desmurget, 2010). As such, the basal ganglia are involved in a number of highly prevalent diseases affecting both movement and cognition, including Parkinson's disease, Huntington's disease, Tourette's syndrome, schizophrenia, addiction, and compulsive disorders (Modell et al., 1989; Carlsson and Carlsson, 1990; DeLong, 1990; Mink, 2001; Albin and Mink, 2006; Belin et al., 2009). The striatum is the largest structure in the basal ganglia and receives most of the input to the basal ganglia from the rest of the brain. One of the most intriguing features of the striatum is the appreciable amount of inhibitory interconnections it contains, although it is currently unknown what role these connections play in the proper function of the striatum. This study examines the functional roles of two forms of intra-striatal inhibition—lateral and feedforward inhibition—using a highly detailed, biophysically accurate, large-scale computational model of the striatal network.

This report focuses on the interactions of two types of striatal cells. Eighty-five to ninety-five percent of the cells within the striatum are medium spiny neurons (MSNs) (O'Donnell and Grace, 1993). These cells comprise the output of the striatum, projecting to downstream structures in the basal ganglia (pallidum and substantia nigra), with extensive axonal arborizations occurring within the striatum as well (Kita and Kitai, 1988). These MSN-MSN connections are GABA-Aergic and are termed lateral inhibition. Of the remaining striatal cells, about half are parvalbumin-containing fast-spiking interneurons (FSIs) (Kawaguchi et al., 1995). FSIs arborize extensively within a locally confined area, inhibiting MSNs via GABA-A receptors (Kita et al., 1990). FSI deficiencies have been linked with dystonia and Tourette syndrome (Gernert et al., 2000; Kalanithi et al., 2005; Berke, 2008; Gittis et al., 2011). Because they receive connections directly from striatal input structures such as cortex, rather than receiving their input from within the striatum, these connections are termed feedforward inhibition (though see Wilson, 2007). Combined, these two intrastriatal circuits are presumed to critically contribute to striatal function, though their specific functional roles are a subject of many investigations (Plenz, 2003; Gurney et al., 2004; Tepper et al., 2008).

The presence of such prevalent lateral inhibitory interconnections between striatal cells has long driven conceptual models of striatal function, and, by extension, basal ganglia function. For example, the existence of so many inhibitory connections between MSNs spurred the long-dominant hypothesis that the striatum functioned as a winner-take-all, competitive neural network, with lateral (MSN-to-MSN) inhibition enabling strongly activated MSNs or MSN ensembles to shut down competing MSNs/MSN ensembles (Groves, 1983; Wickens et al., 1991; Fukai and Tanaka, 1997). Interestingly, ensuing studies found that single MSN inhibitory post-synaptic potentials (IPSPs) are actually very weak and rarely reciprocal (Pennartz and Kitai, 1991; Jaeger et al., 1994; Plenz, 2003). These findings led to other suggestions for the role of lateral inhibition, including the hypotheses that it might facilitate ensemble synchronization by holding the MSN near the relatively depolarized GABA reversal potential (Plenz, 2003), might enhance the coherence of large cellular ensembles (Ponzi and Wickens, 2010, 2012, 2013), or might work in concert with feedforward inhibition to optimize ensemble representation (Yim et al., 2011). To date, a general consensus on the functional role of lateral inhibition in the striatum has yet to emerge.

Likewise, the anatomy of the feedforward inhibitory projection has fueled a number of hypotheses regarding its functionality, though with somewhat less experimental data available for evaluating each theory. The observation that FSI-to-MSN projections form clusters of synapses near the MSN soma and produce relatively large IPSPs in the postsynaptic cell (Kawaguchi et al., 1995; Koos and Tepper, 1999) suggests that FSIs tonically suppress MSN activity, with only strongly activated corticostriatal ensembles able to overcome FSI inhibition (Parthasarathy and Graybiel, 1997; Gage et al., 2010). Another possibility is that tonic FSI spiking may enforce MSN synchronicity by defining narrow interspike time windows in which MSNs may fire (Pouille and Scanziani, 2001), or that FSIs may reset the striatal network by shutting down action representations of cellular ensembles (Wickens and Arbuthnott, 1993; Plenz, 2003; Carrillo-Reid et al., 2008). More recent studies have suggested that while individual FSI-to-MSN IPSPs are powerful, the inhibitory effects of feedforward inhibition are surprisingly subtle, leading to the hypothesis that FSIs precisely control MSN spike timing in order to influence synaptic plasticity in the striatal network (Tepper and Bolam, 2004; Wilson, 2007; Tepper et al., 2008; Urbanczik and Senn, 2009).

Determining the role of inhibition in the striatal network *in vivo* is extremely difficult with current experimental techniques. We therefore studied the functionality of both lateral (MSN-to-MSN) and feedforward (FSI-to-MSN) inhibition using a biophysically constrained, large scale computational model of the striatal network. In an effort to accurately capture the complex dynamics of inhibitory and excitatory inputs in the dendrites of MSNs, we explicitly included dendrites and almost all known ionic channels in both the FSI and MSN models. We found that lateral inhibition enabled large ensembles of synchronously firing MSNs to strongly suppress non-synchronous MSNs. We found that feedforward inhibition effectively suppressed MSN activity—especially non-synchronous MSN activity—but only when FSI cells fired asynchronously. These results suggest that the functional role of lateral inhibition may be to aid MSN ensemble synchronization and formation, while the functional role of feedforward inhibition may be to suppress less active MSN ensembles in favor of more active MSN ensembles. These findings will help to refine and inspire both new and existing conceptual models of the function of the striatum and of the basal ganglia.

## Methods

The model was developed in the NEURON 7 simulation environment (Hines and Carnevale, 1997; Carnevale and Hines, 2005). Simulations were performed in parallel on a 32-node cluster with dual 2.8 GHz processors per node (Apple Computers, Cupertino, CA, USA). Data analysis was performed using MATLAB (Mathworks Inc, Natick, MA). Unless otherwise noted, all simulations were performed using a 2744 MSN, 144 FSI, 280 μm cubic network. In this configuration, a 2-s long simulation of the full network required 30 h to load and 12 h to run.

### Morphology and physiology of the MSN model

The MSN model has been previously described in detail (Wolf et al., 2005), and is available on ModelDB (http://senselab.med.yale.edu/ModelDB/), so we focus only on the most salient aspects of the single cell model in this description. Cell dimensions (dendritic length and diameter, soma size), and passive properties were set to match published values (Wilson, 1992; O'Donnell and Grace, 1993). The MSN model consists of 189 compartments and includes almost all intrinsic currents known to be expressed in the MSN, including: fast (NaF) and persistent sodium (NaP); fast-inactivating (KAf) and slow-inactivating (KAs) A-type, 4-AP-resistant, persistent delayed-rectifying (KRP), and inward-rectifying (KIR) potassium currents; large-conductance (BK) and small-conductance (SK) calcium-dependent potassium currents; N-(CaN), P/Q-(CaP/Q), R-(CaR), and L-type (Cav1.2) high-voltage activated calcium channels; and T-(CaT) and L-type (Cav1.3) low-voltage activated calcium channels. These channels were distributed throughout the cell in accordance with published data when possible. If not known, channels were assumed to be distributed uniformly throughout the cell unless this resulted in non-physiological behavior (see Wolf et al., 2005). All biophysical and kinetic properties (i.e., steady-state parameters and time constants for activation/inactivation) for each channel in the model were taken directly from published data (see Wolf et al., 2005). Channel kinetics and voltage-dependencies from channels isolated in striatal MSN cells were used when available, and supplemented with parameters derived from dorsal striatal cells and other neurons as necessary. The model was tuned solely by balancing the maximum conductance levels of all intrinsic currents against each other to match the response of an *in vitro* cell to current injection (Wolf et al., 2005). Spines were not explicitly modeled, but we accounted for their contribution to membrane area (Segev and Burke, 1998). Each tertiary dendrite was comprised of 11 compartments to ensure spatial accuracy, and inputs were placed in the middle of the appropriate compartment in order to acquire second order correct solutions (Carnevale and Hines, 2005). All model MSN cells utilized the same tuning throughout the network.

The internal calcium concentration in a thin shell just inside the cell membrane was tracked for each compartment. BK and SK currents were regulated by calcium influx via N-, P/Q-, and R-type calcium channels. The remaining calcium currents (1.2 and 1.3 L-type and T-type) contributed to a separate pool that did not regulate the BK and SK currents, in accord with published experimental results (Vilchis et al., 2000).

### Morphology and physiology of the FSI model

The FSI model has been previously described (Kotaleski et al., 2006), and includes fast sodium (Na), two types of delayed-rectifying potassium currents (Kv1.3 and Kv3.1/3.2), and an inactivating potassium current (KA). We reconstructed the model in NEURON and converted it to three dimensions. Importantly, the number of compartments in the model was increased to 148 (d-lambda value of 0.2) to ensure second order spatial resolution in the model (Carnevale and Hines, 2005). Accordingly, the soma was comprised of one compartment, the primary dendrites of three compartments each, the secondary dendrites of five compartments each, and the tertiary dendrites of nine compartments each. Otherwise, the model's morphology, passive properties, and active channels are the same as reported previously (Kotaleski et al., 2006). Each FSI received 84 glutamatergic inputs (AMPA/NMDA pairs) and 84 GABAergic inputs placed throughout the cell. All FSI model cells used the same tuning throughout the model network.

### Network topology

The MSN network was modeled as a cube of MSNs spaced 20 μm apart from each other—giving a spatial density of 88,900 cells per cubic millimeter, to match reported results (Oorschot, 1996; Humphries et al., 2010). FSIs were randomly interspersed throughout the network cube with a uniform probability distribution along the x, y, and z axes. MSNs with somas within 380 μm of each other had a uniform 15.5% unidirectional probability of being connected (Czubayko and Plenz, 2002; Tunstall et al., 2002; Koos et al., 2004; Taverna et al., 2004; Humphries et al., 2010). MSNs with somas within 250 μm of an FSI soma had a uniform 25% probability of receiving a projection from the FSI (Gittis et al., 2010; Humphries et al., 2010). The ratio of FSIs:MSNs was set at 4 FSIs: 90 MSNs to match reported data (Kawaguchi et al., 1995; Luk and Sadikot, 2001; Bolam et al., 2006; Oorschot, 2013). Since the network is intended to be a generalized representation of a small section (0.022 mm^3^) of striatal tissue, we did not differentiate between projections among D1 and D2 expressing cells, though differences in connectivity between these populations have been shown to exist (Taverna et al., 2008; Chuhma et al., 2011).

Lateral projections (MSN to MSN) in the model randomly connect to compartments (uniform probability) in the secondary and tertiary dendrites of MSN to match published results (Wilson and Groves, 1980). MSNs make between 1 and 3 contacts per projection (Scheuss and Neher, 2001)—in the model, if one MSN connects to another, it has an 83% chance of making one contact, a 13% chance of making two contacts, and a 4% chance of making three contacts (percentages obtained from an exponential fit). Reciprocal connections were permitted, but self-connections were not, to match published data (Jaeger et al., 1994). We did not “wrap” projections from cells on one side of the network to the other side, in order to keep the model easily scalable. Because of this, and because the size of the network is approximately the size of one MSN dendritic arbor, neurons in the periphery of the network see fewer inhibitory inputs than would be expected *in vivo* (see Results).

Feedforward projections in the model randomly connect to compartments (uniform probability) in the soma and primary dendrites of an MSN, in agreement with published findings (Kita et al., 1990; Bennett and Bolam, 1994; Bolam et al., 2000). If an FSI connects to an MSN, it forms between 7 and 12 contacts (Koos et al., 2004), with the exact number of contacts randomly chosen with uniform probability. Both lateral and feedforward connections form GABA-A synapses with approximately the same conductance levels (Planert et al., 2010). Lateral and feedforward connections had delays of 2.4 ms to match published results (Tepper et al., 2004).

Cortical inputs to both FSIs and MSNs—consisting of excitatory AMPA/NMDA pairs—were generated according to the algorithm described below. Each input train was independent of other input trains, unless otherwise detailed for a specific experiment. Cortical connections innervated the whole cell for the FSI model (Kotaleski et al., 2006), while they were confined to the dendritic compartments of the MSN to match published findings (Wilson, 1992). Cortical contacts were randomly assigned to compartments with uniform probability.

### Synaptic input generation

As described previously (Wolf et al., 2005; Moyer et al., 2007), explicit glutamatergic and GABAergic synapses were modeled using a two-state synapse with time constants set to published values (Galarreta and Hestrin, 1997; Gotz et al., 1997; Chapman et al., 2003). Each glutamatergic synapse consisted of an AMPA and NMDA pair receiving the same input train. Glutamatergic synapses were placed throughout the dendrites of the MSN, in accordance with published results (Wilson, 1992; Gracy et al., 1999). GABAergic synapses were distributed throughout the MSN but clustered near the soma in agreement with physiological data (Pickel and Heras, 1996; Fujiyama et al., 2000). Both GABAergic and glutamatergic synapses were distributed throughout the FSI (Kotaleski et al., 2006). In the MSN, AMPA and NMDA channels contributed to the calcium pool not associated with the SK/BK currents—10% of NMDA current and 0.5% of AMPA current were designated as calcium current as has been described in previous studies (Burnashev et al., 1995). AMPA (Myme et al., 2003), NMDA (Dalby and Mody, 2003), and GABA (Nusser et al., 1998) conductance levels were set to published values (see Wolf et al., 2005).

Synaptic inputs were modeled using the NetStim object provided in the NEURON package. Each synapse (AMPA/NMDA or GABA) received an independent spike train. Each spike train was generated using the following algorithm (see Wolf et al., 2005): first, a constant interspike interval (ISI) train was generated at the desired frequency. Each spike was then pulled anew from a Gaussian distribution centered at the original spike time. The resulting train was then randomly shifted, and this process was repeated for each of the synapses. Uncorrelated input was generated by using a large shift (one ISI) and a large standard deviation (1/4 of the ISI). Using this algorithm, as opposed to a standard Poisson process, allowed us to generate partially synchronized, though still randomized, input trains. The ratio of glutamatergic inputs to GABA inputs for FSIs was held constant at roughly 1:1 for all simulations (Blackwell et al., 2003). The synaptic input frequency is calculated as the summed number of glutamatergic and GABAergic inputs per second.

The traces shown in **Figures 3A,B** are representative samples from the network simulations shown in **Figure 5**. Inhibitory postsynaptic potential (IPSP) amplitudes and time courses for lateral and feedforward inhibition shown in **Figures 3C–F** were obtained by subtracting MSN membrane voltage traces from a 512 MSN network simulation with lateral (feedforward) inhibition active from the same network simulation without lateral (feedforward) inhibition active. To simplify the calculation, only one MSN (FSI) received enough depolarizing input to spike, while all other MSNs received unique synaptic input trains that placed them at various potentials below firing threshold. Only data from the first spike in the simulation was used for the plot. For each IPSP, we found the maximum and minimum value of the IPSP, and then used whichever value (max or min) had a greater absolute value. Therefore, there are no IPSPs of zero amplitude, or very small absolute value, on the plot.

Synaptic input frequencies in **Figure 4** are calculated for glutamatergic inputs only. For **Figure 4A**, FSIs in the network received glutamatergic synaptic inputs at a frequency of 300 Hz. For **Figure 4B**, MSNs in the network received glutamatergic synaptic inputs at a frequency of 1000 Hz.

## Results

### Cell models

**Figure 1:**
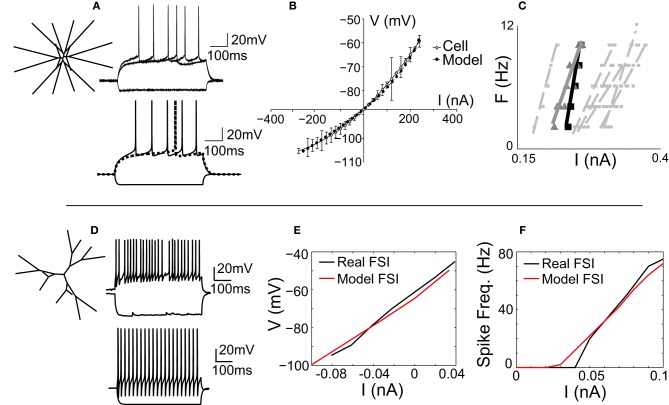
**The MSN and FSI models match *in vitro* cellular data**. **(A)** Left, the medium spiny neuron (MSN) model is a stylized three-dimensional representation of an adult rat nucleus accumbens MSN (Wolf et al., 2005). Bottom, the response of the MSN model to current injection matches the response of *in vitro* MSN to current injection (top). **(B)** The model's voltage-current (V-I) response (gray line) matches the V-I response of an *in vitro* MSN (black line). **(C)** The spike frequency vs. current (F-I) response of the model (black line) matches the F-I response of an *in vitro* cell (gray line) and is representative of other *in vitro* MSN cells (dashed gray lines). **(D)** Left, the fast-spiking interneuron (FSI) model is a stylized three dimensional representation of an adult rat dorsal striatal FSI (Kotaleski et al., 2006). Bottom, the response of the FSI model to current injection is tuned to represent the response of an *in vitro* FSI to current injection (top). **(E)** The V-I response of the FSI model (red line) matches the V-I response of an *in vitro* FSI (black line). **(F)** The F-I response of the FSI model (red line) matches the F-I response of an *in vitro* FSI (black line).

The MSNmodel is a stylized representation of a nucleus accumbens core MSN, which has been previously described (Wolf et al., 2005; Moyer et al., 2007). It consists of 189 compartments and includes almost all currents known to be expressed in the MSN. For this study, the morphology of the MSN model was adjusted so that the model's dendritic arbor filled a three-dimensional cube, rather than a two-dimensional square (Figure 1A, inset). As reported previously (Wolf et al., 2005), the MSN model was tuned to match spike shape (Figure 1A), voltage-current response (Figure 1B), and spike frequency behavior (Figure 1C) of an *in vitro* MSN from an adult (P-X) rat accumbens core neuron.

The fast-spiking interneuron (FSI) model was adapted from a previously published model of a rat dorsal striatal parvalbumin-expressing FSI (Kotaleski et al., 2006). Channel parameters, including conductance levels, are as reported previously (Kotaleski et al., 2006). The morphology of this model FSI is maintained in our version of the FSI, with two exceptions: the cell is three-dimensional (Figure 1D, inset), and the number of compartments is increased to ensure spatial accuracy (see Methods). The model was tuned to match the spike shape (Figure 1D), voltage-current relationship (Figure 1E), and spike frequency-current (F-I; Figure 1F) response of an *in vitro* FSI.

### Network topology

**Figure 2:**
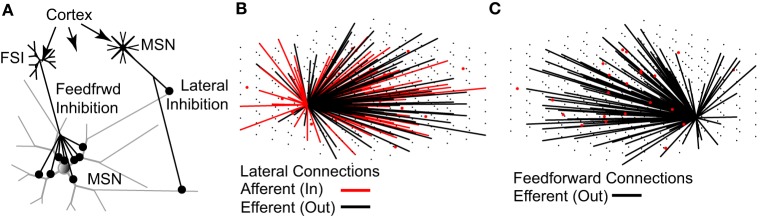
**The topology of the striatal network model accurately represents physiological data**. **(A)** Lateral (MSN-to-MSN) inhibitory connections contact distal portions of the target MSN and make 1–3 contacts. Feedforward (FSI-to-MSN) inhibitory connections contact proximal regions of the target MSN and make 7–12 contacts. Both FSIs and MSNs receive excitatory glutamatergic inputs from the cortex and other regions. **(B)** MSNs (black dots) have a 15.5% probability of sending an efferent (black lines) connection to each of the other MSNs in the network, and a 15.5% probability of receiving an afferent (red line) connection from each of the other MSNs, provided the MSN somas are within 380 μm of each other. **(C)** FSIs (red dots) have a 25% chance of sending an efferent (black lines) connection to each of the MSNs in the network, provided the MSN soma is within 250 μm of the FSI soma. As noted throughout the text, the network topology is strictly based on previously published anatomical observations.

The topology of the model network (Figure 2) is based strictly on previously published studies (see Methods). As such, the network represents a 0.022 mm^3^ cube of striatal tissue, 280 um per side—approximately the size of one MSN dendritic arbor (Wilson and Groves, 1980; Wilson, 1992). On average, each MSN received 636 lateral inhibitory synaptic connections from 430 other MSNs and 116 feedforward inhibitory synaptic connections from 18 FSIs. Accordingly, each MSN receives one-third to one-half the expected number of lateral inhibitory input connections [range estimated to be 1200–1800 lateral connections per MSN (Wilson and Groves, 1980; Wilson, 2007)] but approximately the correct number of feedforward inhibitory input connections [range estimated to be 50–175 feedforward connections per MSN (Tepper et al., 2004; Wilson, 2007)].

### Comparison of the network to physiology

**Figure 3:**
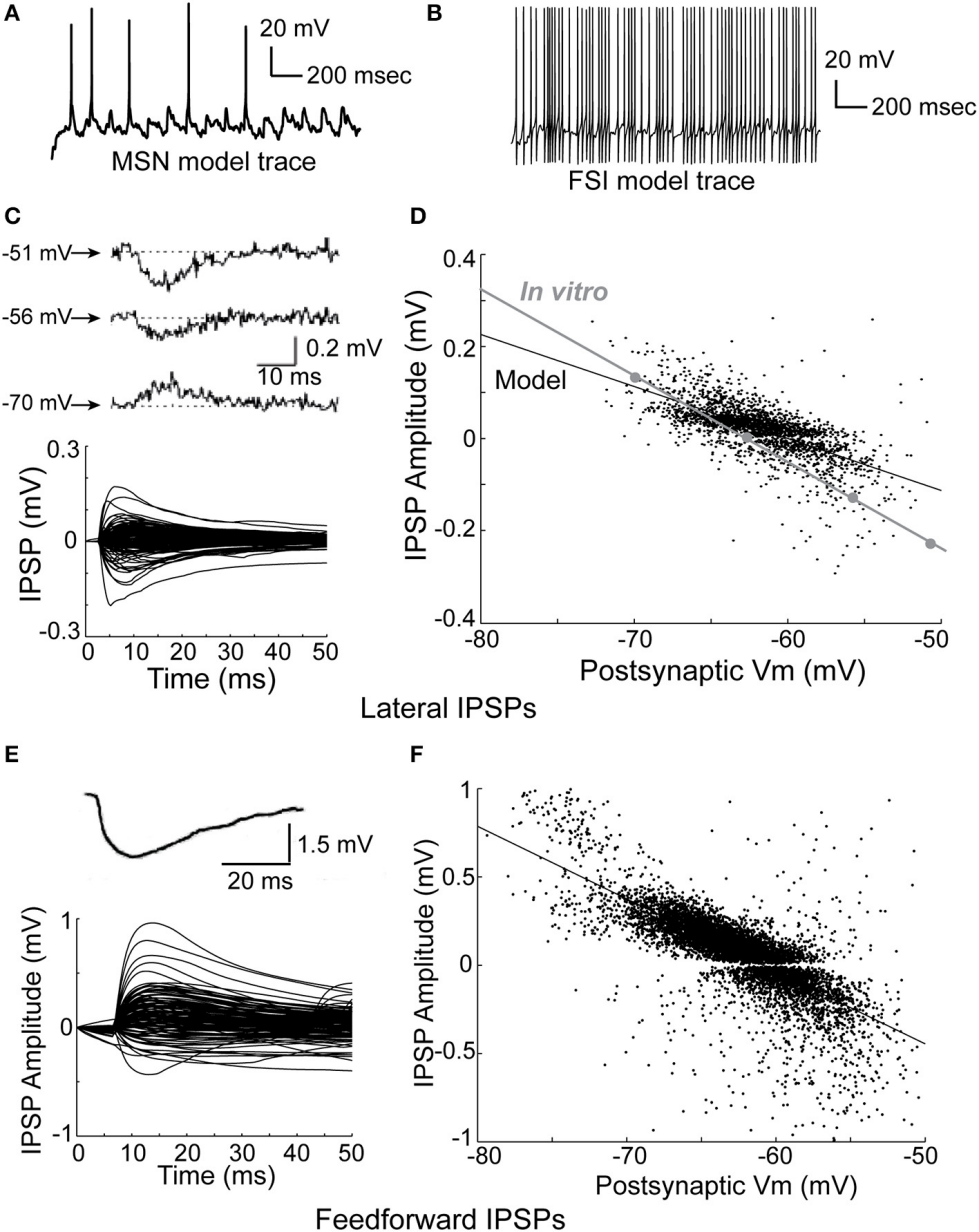
**Physiology of the network model**. **(A)** Intracellular voltage trace of a representative MSN model cell during a network simulation. **(B)** Intracellular voltage trace of a representative FSI model cell during a network simulation. **(C)** Bottom, the amplitude and time course of lateral (MSN-to-MSN) IPSPs in the model compare favorably to the amplitude and time course of *in vitro* lateral IPSPs (top; taken from Tunstall et al., 2002). **(D)** Lateral IPSP amplitudes were in the range ±0.2 mV, matching published *in vitro* data. The dependence of lateral IPSP amplitude on postsynaptic membrane voltage in the model closely approximates *in vitro* data (inset; adapted from Tunstall et al., 2002). **(E)** Bottom, the amplitude and time course of feedforward inhibitory IPSPs in the model approximates *in vitro* data (top; taken from Tepper et al., 2004). **(F)** Feedforward IPSP amplitudes are roughly five times larger (range ±1 mV) than lateral IPSP amplitudes, approximating *in vitro* results. Taken together, these results indicate that inhibition in the network model is consistent with published *in vitro* data, and demonstrate that IPSPs in the model are highly dependent on voltage–and time-dependent interactions in the dendrites of the model cells.

The model network is able to accurately reproduce observed physiological phenomena, such as IPSP size, time course, and dependence on membrane voltage for both lateral inhibition (Figures 3C,D), and feedforward inhibition (Figures 3E,F). Importantly, previous studies have reported that FSI inputs to MSNs should be approximately 4–10 times larger than MSN inputs to MSNs (Tepper et al., 2004)—in the model, FSI inputs are 4–8 times larger than MSN inputs. Accordingly, both lateral and feedforward IPSPs are accurately reproduced in the model.

We fit a double exponential to each IPSP using the form V = exp(−t/T1) − exp(−t/T2), where V is voltage, t is time, and T1 and T2 are time constants, and used a one-sample z-test to compare the *in vitro* data to the model data, where a *z*-value in the range of (−1.96, 1.96) indicates 95% confidence in equivalence. After discarding any fits with an R-squared value of less than 80%, this gave a mean T1 of 10.4 ms (*SD* of 3.9 ms) and a mean T2 of 10.2 ms (*SD* of 3.6 ms) for lateral inhibition in the model. For comparison, the *in vitro* data in Figure 3 gave time constants of 10.5 ms (*z* = −0.18), 10.2 ms (*z* = 0.35), and 10.5 ms (*z* = −0.18) for T1, and 6.3 (*z* = 7.4), 7.3 (*z* = 5.5), and 8.1 ms (*z* = 4.0) for T2. For feedforward inhibition, fitting the model gave a mean T1 of 6.5 ms (*SD* of 3.7 ms), and a mean T2 of 4.4 ms (*SD* of 4.9 ms). The *in vitro* data in Figure 3 gave a time constant of 9.9 ms (*z* = −6.3) for T1 and 5.7 (*z* = −1.8) ms for T2. Therefore, the model's values for lateral T1 and feedforward T2 time constants match *in vitro* data, while the model's values for lateral T2 and feedforward T1 time constants do not.

### Relative effects of lateral and feedforward inhibition on unstructured MSN spiking

**Figure 4:**
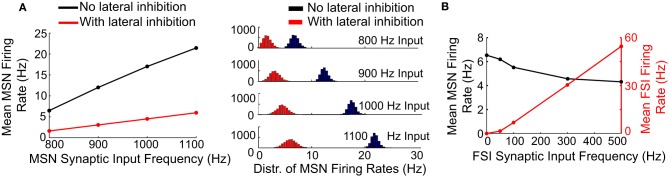
**Effects of lateral and feedforward inhibition on MSN spiking in the model**. **(A)** Left, with lateral inhibition active (red line), the mean firing rate of MSNs in the network is significantly reduced compared to the case when lateral inhibition is inactive (black line). Right, with lateral inhibition active (red histograms), the standard deviation of the distribution of MSN firing rates in the network is increased compared to the case when lateral inhibition is inactive (black histograms). These effects become more pronounced at higher MSN spike rates. **(B)** Feedforward inhibition only slightly suppresses MSN spike firing in the model (black line), even at high FSI spike frequencies (red line). Unlike lateral inhibition, feedforward inhibition does not affect the distribution of MSN firing rates in the network (data not shown). These results indicate that lateral inhibition has a powerful effect on MSN spiking in the model relative to feedforward inhibition, despite the fact that cell-to-cell connections are weaker for lateral inhibition.

We examined the effects of lateral (MSN-to-MSN) and feedforward (FSI-to-MSN) inhibition on MSN firing rate in response to increasing frequencies of unstructured synaptic inputs (Figure 4A). Lateral inhibition had a significant effect on the mean MSN spike rate in the network, progressively decreasing spiking by up to 70% for input frequencies between 800 and 1100 Hz (Figure 4A, left). Lateral inhibition also increased the standard deviation of the distribution of MSN firing rates as the input frequency to the MSNs increased (Figure 4A, right), in agreement with previous findings (Humphries et al., 2010; Ponzi and Wickens, 2010; Yim et al., 2011). In contrast, feedforward (FSI-to-MSN) inhibition reduced mean MSN spiking by only 33%, from 6.5 to 4.3 Hz (Figure 4B, black trace), with an FSI firing rate of 55 Hz. Feedforward inhibition did not affect the distribution of MSN firing rates (data not shown).

Accordingly, lateral inhibition appears to be capable of significantly limiting uncorrelated spiking in the striatal network model, despite the fact that MSNs form few connections per cell and contact mostly distal locations on the target MSN. In contrast, feedforward inhibition is relatively incapable of suppressing uncorrelated MSN firing, despite the fact that FSIs form multiple proximal connections with target MSNs.

### Effects of lateral inhibition on striatal neural ensembles

**Figure 5:**
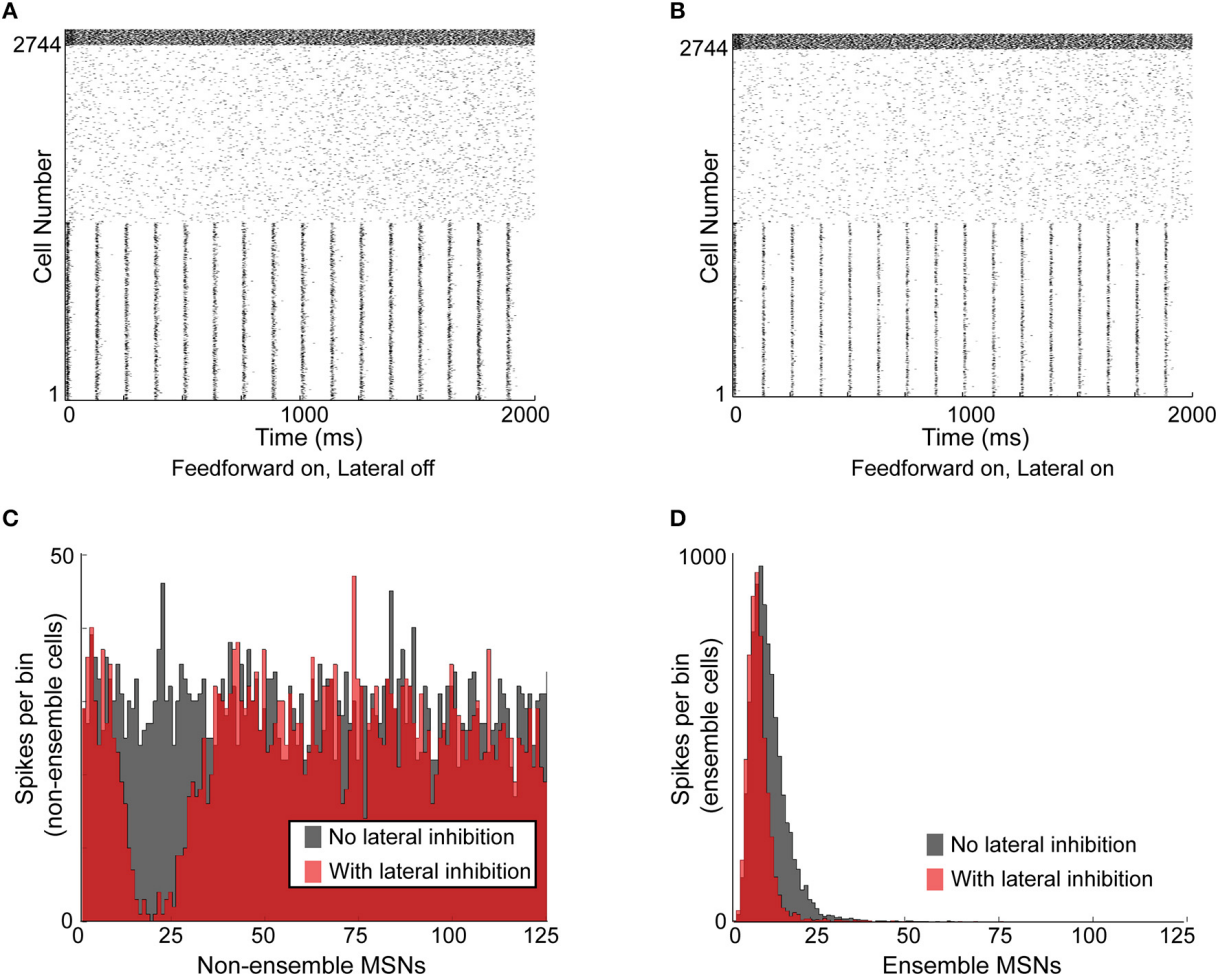
**Functional effects of lateral inhibition in the model**. **(A)** Raster plot of the network with lateral inhibition inactive. MSNs are cells number 1–2744, FSIs are cells 2745–2888. MSNs 1–1372 are entrained to a structured 8 Hz rhythm, while MSNs 1373–2744 are responding to noisy, unstructured input. FSIs are responding to noisy, unstructured input. **(B)** Raster plot of the same network with lateral inhibition active. **(C)** Histogram of non-ensemble MSNs (cells 1373–2744) aligned to each ensemble spike in MSNs 1–1372. Comparing the case with lateral inhibition inactive (gray histogram) to the case with lateral inhibition active (red histogram), it is clear that lateral inhibition significantly suppresses spiking in the non-ensemble MSNs within 5–30 ms of the ensemble spike. **(D)** Histogram of ensemble cells (MSNs 1–1372) aligned to the 8-Hz synchronous input preceding each ensemble spike in MSNs 1–1372. Comparing the case with lateral inhibition inactive (gray histogram) to the case with lateral inhibition active (red histogram), it is clear that lateral inhibition suppresses MSN spikes that occur later in the ensemble response, without affecting MSN spikes that occur early in the ensemble response. Accordingly, lateral inhibition enhances the signal-to-noise ratio of responding MSNs by suppressing non-ensemble MSNs and sharpening the response of ensemble MSNs.

We next examined the effect of lateral inhibition on MSN neural ensembles responding to correlated and uncorrelated inputs. Specifically, we created a network simulation in which half of the MSNs (cells 1–1372) were responding as a partially synchronized ensemble and the other half (cells 1373–2744) were firing randomly (Figures 5A,B). Ensemble cells received a combination of distinct noisy synaptic inputs and shared, precisely timed, 8 Hz rhythmic inputs—representing theta-coordinated input from upstream cortical or limbic structures (Berke et al., 2004). Non-ensemble MSNs each received distinct noisy inputs. There was no difference in connectivity between intra-ensemble MSNs and non-ensemble MSNs. FSIs in the network each received a distinct, noisy 600 Hz input train. By running the simulation with and without lateral inhibition active, with the same connectivity and same set of synaptic inputs, we were able to carefully investigate the effects of lateral inhibition on ensembles in the network.

Upon comparing the activity in the network without lateral inhibition (Figure 5A) and the activity in the network with lateral inhibition (Figure 5B), two observations were immediately apparent. First, each “ensemble spike,” consisting of the population of synchronized MSN cells, significantly suppressed firing in the non-ensemble cells within a window of 5–30 ms following the ensemble spike (Figure 5C). Second, lateral inhibition narrowed the response histogram of the ensemble cells in response to structured input, suppressing intra-ensemble spikes that occurred later in the response (Figure 5D). This decreased the standard deviation of the ensemble spike from 5.15 to 4.43 ms without reducing the maximum amplitude. An additional simulation using a smaller, 512 MSN network is included as Supplemental Figure 1, demonstrating that these results are not dependent on the specific network setup used for Figure 5.

Therefore, lateral inhibition improves the timing precision of the ensemble response to coordinated input by suppressing the activity of MSNs (both intra-ensemble cells and non-ensemble cells) that are not precisely synchronized with the active ensemble.

### Effect of feedforward inhibition on striatal neural ensembles

**Figure 6:**
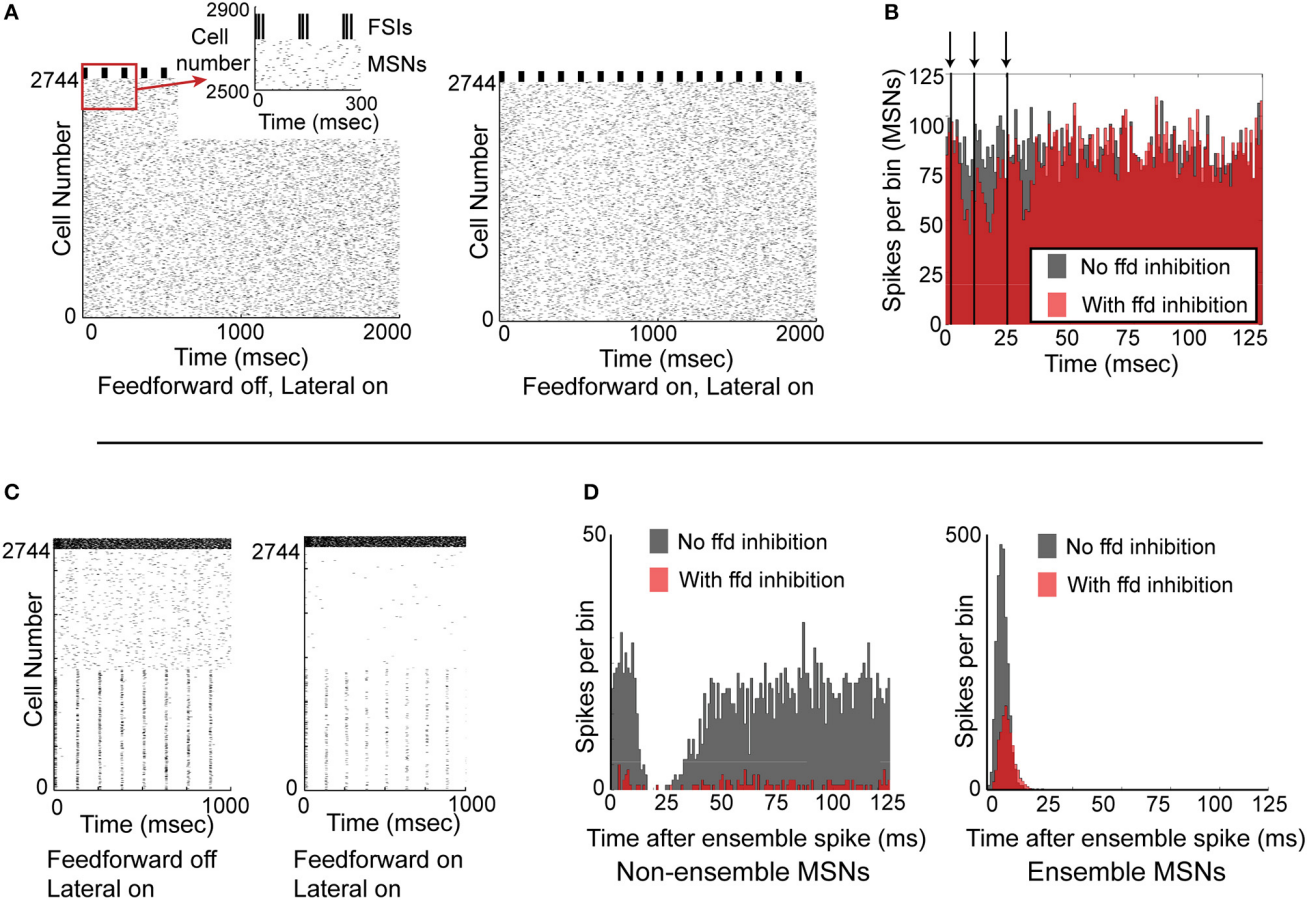
**Functional effects of feedforward inhibition in the model**. **(A)** Raster plot of the network with feedforward inhibition inactive (left) and with feedforward inhibition active (right). MSNs are cells number 1–2744, FSIs are cells 2745–2888. MSNs are responding to noisy, unstructured input, while FSIs are completely synchronized and bursting every 125 ms. **(B)** Histogram of the 2744 MSNs aligned to the beginning of the FSI bursts. With FSIs completely synchronized, feedforward inhibition only mildly suppresses MSN spiking. Arrows and vertical black lines indicate FSI spikes. **(C)** Raster plot of network with feedforward inhibition inactive (left) and with feedforward inhibition active (right). In this simulation, MSNs 1–1372 are responding to structured input, while MSNs 1373–2744 are responding to unstructured input. FSIs are desynchronized and are responding with a mean spike rate of 60 Hz. **(D)** Left, histogram of non-ensemble cells (MSNs 1373–2744) aligned to each ensemble spike in cells 1–1372. Comparing the case with feedforward inhibition inactive (gray histogram) to the case with feedforward inhibition active (red histogram), it is clear that feedforward inhibition can significantly suppress non-coordinated MSN spiking. Right, histogram of the ensemble cells (MSNs 1–1372) aligned to each ensemble spike. Comparing the case with feedforward inhibition inactive (gray histogram) to the case with feedforward inhibition active (red histogram), it is clear that feedforward inhibition suppresses but does not eliminate coordinated MSN spiking. Accordingly, feedforward inhibition suppresses both coordinated and uncoordinated MSN spiking, though it is unable to completely suppress either type of activity.

We next investigated whether synchronized feedforward inhibition could similarly suppress MSN activity. We created a network simulation in which all 2744 MSNs received noisy synaptic input, and all 121 FSIs received precisely the same synaptic input and therefore spiked in perfectly synchronized approximately 60 Hz bursts every 125 ms. Comparing the activity of the network without feedforward inhibition (Figure 6A, left) to the activity of the network with feedforward inhibition (Figure 6A, right), it is apparent that synchronous feedforward inhibition does not significantly suppress MSN firing. Binning the spike times of the MSNs in a histogram aligned to the beginning of every FSI burst (Figure 6B) makes it clear that while perfectly synchronized feedforward inhibition can suppress some MSN activity, it does not strongly suppress MSN firing, even when the MSNs receive noisy, uncorrelated inputs.

Since synchronized feedforward inhibition does not appear to significantly suppress MSN firing, we asked whether desynchronized feedforward inhibition might be more effective in suppressing MSN activity. To study this, we created a network simulation in which half the MSNs responded as a partially synchronized ensemble (receiving a combination of noisy inputs and structured, 8 Hz inputs) while the other half of the MSNs were not synchronized at all—receiving only noisy synaptic inputs (Figure 6C). Comparing the activity of the network without (Figure 6C, left) and with feedforward inhibition active (Figure 6C, right), it was apparent that feedforward inhibition suppressed both MSN ensemble and non-ensemble activity. Binning the MSN spikes in a histogram aligned to the 8 Hz MSN ensemble spikes (Figure 6D) clarifies that while non-ensemble MSN spikes were strongly suppressed (12.6% of spikes remained), ensemble MSNs were only moderately suppressed (42.8% of spikes remained). An additional simulation using a smaller, 512 MSN network is included as Supplemental Figure 2, demonstrating that these results are not dependent on the specific network setup used for Figure 6.

Accordingly, feedforward inhibition appears to disproportionately limit non-synchronously firing MSNs in favor of synchronously firing MSNs, and appears to act more effectively when FSIs are desynchronized.

## Discussion

### Accuracy of the model and comparison with other models

To our knowledge, this model is the most accurate representation to date of the current state of knowledge of the connectivity and parameters of the striatal network. It is also the first striatal network model to incorporate multi-compartment MSN and FSI models (Wolf et al., 2005; Kotaleski et al., 2006) containing multiple species of ionic currents and biophysically detailed dendrites. The model captures several important aspects of striatal function, including intrinsic MSN and fast-spiking interneuron (FSI) cellular function (Figure 1), network topology (Figure 2), and voltage–and time-dependent characteristics (Figure 3) of the lateral (MSN-to-MSN) and feedforward (FSI-to-MSN) inhibitory connections.

Our operating philosophy in building the single cell MSN model, and later the network model, was to include as much experimentally verified data as possible in order to remain objective. A reduced computational model may accurately reproduce some, perhaps most, of the behavior we present here. However, it is only possible to conclude this with reasonable certainty *after* having built and analyzed this more complicated network containing representative neurons with all known currents and parameters. Future studies using this network will allow for a more thorough examination of the parameter space of the model network, including the construction of the network and projection topography between cells, as well potential comparisons to an optimized version of the network using simplified cells. In a complex network such as the one modeled here, it is important to confirm that any observed effects are not a result of the specific network setup. We did so by using a single seed value for the random number generator used to build the network—including the network connections, FSI positioning, and synaptic input timing. While we did not have the resources to perform a full statistical analysis of the network output, by performing a large number of simulations with different seed values, we confirmed that the results here are robust (see Supplemental Material for examples).

Importantly, we did not explicitly account for gap junctions between FSIs in the model, though these have been observed experimentally (Kita et al., 1990; Koos and Tepper, 1999; Galarreta and Hestrin, 2001; Fukuda, 2009). We did, however, account for the effects of gap junctions on FSI function and feedforward inhibition, in the sense that gap junctions have been shown to synchronize FSIs in computational modeling studies (Hjorth et al., 2009; Lau et al., 2010; Klaus et al., 2011), and we simulated the extreme condition in which FSIs were completely synchronized with each other (Figures 6A,B). As noted, we found that feedforward inhibition was actually less effective when FSIs were synchronized, suggesting further work is necessary to define the role of FSI gap junctions with regards to striatal information processing.

Several other groups have created large computational models of the striatum using single compartment cells in order to study the functional effects of the lateral and feedforward inhibitory projections. Using large scale network models consisting of 100–4000 single compartment neurons, researchers have shown that lateral inhibitory interactions in the striatal network enhance the ability of MSNs to express a diverse array of spiking characteristics and form large cellular ensembles with other MSNs (Humphries et al., 2009, 2010; Ponzi and Wickens, 2010, 2012, 2013; McCarthy et al., 2011; Yim et al., 2011). Our findings do not contradict the findings of these studies—however, it is important to note that in general, we examined the effects of inhibition on a shorter time scale (less than 2 s), while in some cases, these studies examined inhibitory interactions over several seconds. Interestingly, other studies have indicated that cellular ensembles may arise intrinsically within the striatum, especially over longer timescales (Carrillo-Reid et al., 2008; Ponzi and Wickens, 2010, 2012; McCarthy et al., 2011). While our study focused on the role of local inhibition during ensemble responses to correlated inputs at millisecond timescales, our conclusions may also apply for cellular ensembles that arise intrinsically.

With respect to feedforward inhibition, a previous model found that feedforward inhibition actually increased MSN spiking (Humphries et al., 2009, 2010). We did not observe this effect. The hypothesis that feedforward inhibition might actually facilitate MSN spiking relies on the observation that the GABA reversal potential (−60 mV in our model) is quite close to the “up-state” potential (Plenz, 2003; Flores-Barrera et al., 2009). That said, the feedforward inputs to the MSN would presumably need to be carefully timed to arrive while the MSN was hyperpolarized yet subside prior to the MSN spiking in response to input. We made no provisions for such timing in our model—accordingly, we cannot rule out that this may occur under certain conditions in the network.

### Functional effects of lateral and feedforward inhibition

The primary reason that lateral inhibition is able to more powerfully suppress MSN firing than feedforward inhibition is because of the significantly greater number of lateral inhibitory inputs per MSN. Additionally, we suggest that the dendritic localization of lateral inhibitory inputs may actually be advantageous for influencing MSN activity. In previous work, we demonstrated that the MSN model's dendrites integrate input independently and together “pull” the soma up toward the spike threshold (Wolf et al., 2005)—meaning that the output of the MSN is almost entirely determined by the input integration of its independent dendrites. Therefore, distally located inhibitory inputs, such as lateral inhibitory inputs, are optimally positioned to regulate dendritic integration and therefore the output of the cell as a whole (Wilson, 2007; Tepper et al., 2008). Our model is unique in that it captures the interaction of these inputs in explicitly modeled dendrites.

A surprising finding of our study is that asynchronous FSI activity appears to have a more profound inhibitory effect on MSN spiking than synchronous FSI activity. Since each feedforward IPSP is already relatively large, we speculate that several FSIs firing in synchrony have only a small reinforcing effect. Given the brief feedforward IPSP time course of ~10 ms, asynchronous spiking would distribute the inhibitory effect of the FSI spiking over a longer time course, and induce more of a sustained suppression than synchronous FSI spiking. Importantly, experimental recordings have observed that FSIs are tonically active *in vivo* (Berke, 2011) and tend to modulate their spiking activity in a coordinated manner, yet are not precisely synchronous (Berke, 2008, 2011). This suggests that asynchronous FSI spiking exerts a more effective inhibitory influence on MSNs *in vivo*, which is supported by our model.

We have demonstrated that lateral inhibition can significantly reduce uncorrelated MSN spiking as well as enable synchronously firing MSNs to strongly suppress non-synchronized MSNs. These findings are in line with the concept that the striatum functions as a competitive neural network, but suggest that it is not only the size of an MSN ensemble which determines the winner in the competition, but also the latency with which the ensemble responds to inputs. The ability of lateral inhibition to significantly constrain uncorrelated activity in the network (Figure 4) also suggests that lateral inhibition plays a role in gain control of the network, limiting network output levels even as the input to the network increases significantly.

Additionally, we suggest that the precise MSN spike timing conveyed by lateral inhibition is important for regulating synaptic plasticity—specifically, dopamine-dependent, spike-timing dependent plasticity. Corticostriatal plasticity has been shown to be driven by a type of spike-timing dependent plasticity in which the presence or absence of dopamine determines whether a synaptic connection strengthens or weakens (Wickens et al., 2003; Goto and Grace, 2005; Lindskog et al., 2006). This type of plasticity is critically dependent on the timing of a spike relative to its inputs. In this light, lateral inhibition precisely defines a narrow temporal window during which MSNs will respond to incoming cortical input, which would help to quickly and accurately define or dissolve neural ensembles.

In contrast, we showed that feedforward inhibition can disproportionately reduce spiking in non-synchronously firing MSNs while sparing synchronously firing MSNs. Recent studies have shown that even though FSIs fire non-synchronously (Berke, 2008), they do show a coordinated increase in firing at the moment of left-right choice in a lever-pressing task (Gage et al., 2010). Additionally, *in vitro* experiments have shown that FSIs burst at the beginning and ending of MSN ensemble formation (Carrillo-Reid et al., 2008). Assuming that synchronously firing MSN neural ensembles represent specific action choices an organism may make in a given situation, and that more active ensembles represent more optimal choices, feedforward inhibition would suppress suboptimal actions in favor of preferred ones. Further, feedforward inhibition would also facilitate switching from one activity to another (Berke, 2011).

Both forms of inhibition would increase the signal-to-noise ratio of MSN ensembles. Lateral inhibition, by enforcing precise synchronization of active MSN ensembles while suppressing non-synchronized cells, would increase the clarity of activated ensembles relative to background activity in the striatum and facilitate signal readout by the pallidum and other downstream structures. Feedforward inhibition would comprise a simple yet effective mechanism for turning off weak neural ensembles while sparing stronger ones, again increasing the clarity of signal presentation in the striatum.

### Conclusion—implications for models of striatal function

Taken together, our results suggest a conceptual framework within which models of striatal and basal ganglia function may be considered. For example, within the context of action selection, where an organism must choose from among several potentially conflicting choices, lateral inhibition would be expected to improve the ability of the network to learn new action representations, to efficiently select only one action at any given time, and to associate the outcome of an action with the correct choice representation. Feedforward inhibition would be expected to ensure the selection of the most appropriate action, to help the striatal network shift between actions, and to prevent multiple action representations from being active simultaneously. Importantly, subjects with impaired lateral inhibition would be expected to exhibit learning deficits as well as impairments in action initiation and control, as with chorea in Huntington's disease and akinesia and cognitive deficits in Parkinson's disease. Subjects with impaired feedforward inhibition would be expected to exhibit deficits in the selection of appropriate actions, as observed in Tourette's disease and obsessive-compulsive disorder. Continued research with animal models of these disorders, along with selective inactivation of either form of inhibition, will allow for the testing of the predictions generated from the comprehensive network model presented here.

### Conflict of interest statement

The authors declare that the research was conducted in the absence of any commercial or financial relationships that could be construed as a potential conflict of interest.

## Supplementary material

Supplemental Figure 1 **Functional effects of lateral inhibition in a 512 MSN version of the model using a different seed value than in Figure 5. (A)** Raster plot of the network with lateral inhibition inactive. MSNs are cells 1–512, FSIs are cells 513–535. **(B)** Raster plot of the same network with lateral inhibition active. **(C)** Histogram of non-ensemble MSNs (cells 257–512) aligned to each ensemble spike in MSNs 1–256. **(D)** Histogram of ensemble cells (MSNs 1–256) aligned to the 8-Hz synchronous input preceding each ensemble spike. As in Figure 5, lateral inhibition suppresses non-synchronous spikes in both the ensemble and non-ensemble cells.

Supplemental Figure 2 **Functional effects of feedforward inhibition in a 512 MSN version of the model using a different seed value than in Figure 6. (A)** Raster plot of the network with FSIs completely synchronized, MSNs desynchronized, and feedforward inhibition inactive (left), and active (right). MSNs are cells 1–512, FSIs are cells 513–535. **(B)** Histogram of the MSN cells in **(A)** aligned to the beginning of each FSI burst. **(C)** Raster plot of the network with FSIs desynchronized, MSNs synchronized, and feedforward inhibition inactive (left) and active (right). **(D)** Histogram of the MSN cells in **(C)** aligned to the beginning of each 8-Hz MSN theta cycle. **(E)** Raster plot of the network with FSIs desynchronized, MSNs desynchronized, and feedforward inhibition inactive (left) and active (right). **(F)** Histogram of the MSN cells in **(E)** binned in 125 ms intervals. As in Figure 6, feedforward inhibition is more effective when FSIs are desynchronized, and feedforward inhibition strongly suppresses non-ensemble MSNs but only moderately suppresses ensemble MSNs.
