# Supplementary figures and images for: Lateral and feedforward inhibition suppress asynchronous activity in a large, biophysically-detailed computational model of the striatal network
Source: Front Comput Neurosci. 2014 Nov 25;8:152. doi: 10.3389/fncom.2014.00152 (PMC4243567; doi:10.3389/fncom.2014.00152)

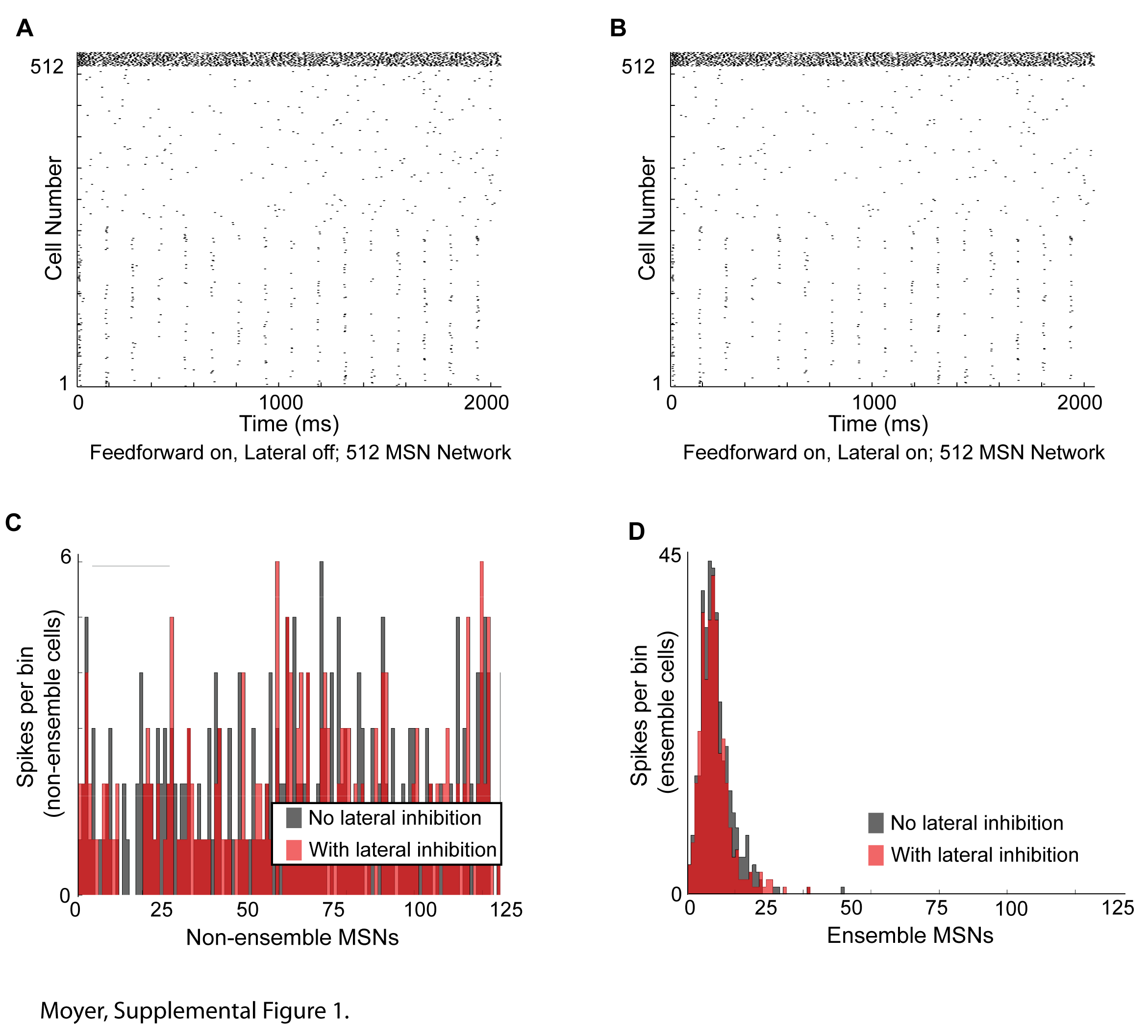

Supplement: Supplemental Figure 1 — Functional effects of lateral inhibition in a 512 MSN version of the model using a different seed value than in Figure 5. (A) Raster plot of the network with lateral inhibition inactive. MSNs are cells 1–512, FSIs are cells 513–535. (B) Raster plot of the same network with lateral inhibition active. (C) Histogram of non-ensemble MSNs (cells 257–512) aligned to each ensemble spike in MSNs 1–256. (D) Histogram of ensemble cells (MSNs 1–256) aligned to the 8-Hz synchronous input preceding each ensemble spike. As in Figure 5, lateral inhibition suppresses non-synchronous spikes in both the ensemble and non-ensemble cells. [file Image1.TIF]

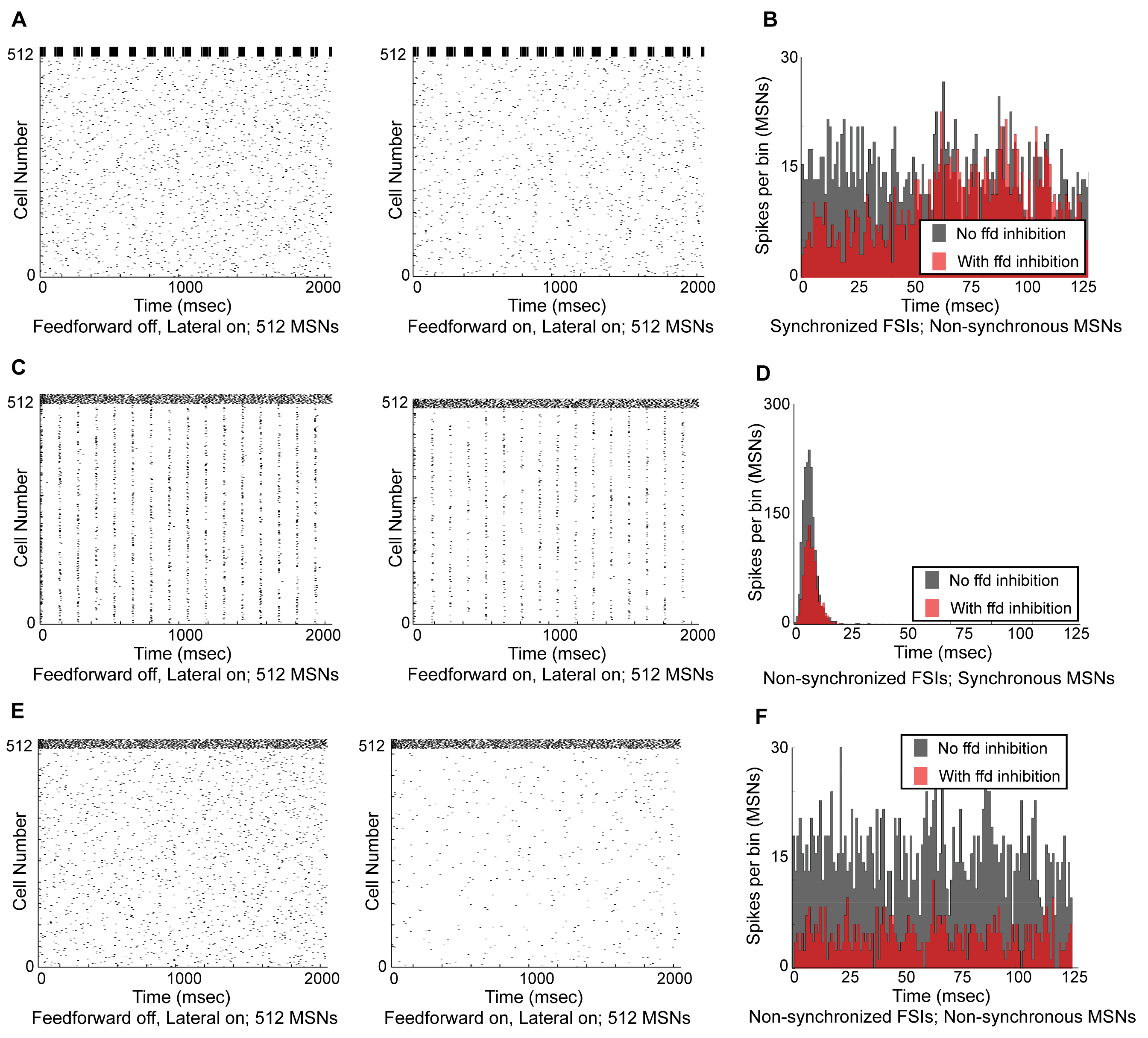

Supplement: Supplemental Figure 2 — Functional effects of feedforward inhibition in a 512 MSN version of the model using a different seed value than in Figure 6. (A) Raster plot of the network with FSIs completely synchronized, MSNs desynchronized, and feedforward inhibition inactive (left), and active (right). MSNs are cells 1–512, FSIs are cells 513–535. (B) Histogram of the MSN cells in (A) aligned to the beginning of each FSI burst. (C) Raster plot of the network with FSIs desynchronized, MSNs synchronized, and feedforward inhibition inactive (left) and active (right). (D) Histogram of the MSN cells in (C) aligned to the beginning of each 8-Hz MSN theta cycle. (E) Raster plot of the network with FSIs desynchronized, MSNs desynchronized, and feedforward inhibition inactive (left) and active (right). (F) Histogram of the MSN cells in (E) binned in 125 ms intervals. As in Figure 6, feedforward inhibition is more effective when FSIs are desynchronized, and feedforward inhibition strongly suppresses non-ensemble MSNs but only moderately suppresses ensemble MSNs. [file Image2.TIF]
